# ISG15 and ISGylation in Human Diseases

**DOI:** 10.3390/cells11030538

**Published:** 2022-02-04

**Authors:** Oygul Mirzalieva, Meredith Juncker, Joshua Schwartzenburg, Shyamal Desai

**Affiliations:** Department of Biochemistry and Molecular Biology, LSUHSC-School of Medicine, 1901 Perdido Street, New Orleans, LA 70112, USA; omirza@lsuhsc.edu (O.M.); majuncker@gmail.com (M.J.); jschw4@lsuhsc.edu (J.S.)

**Keywords:** ubiquitin, ISG15, ISGylation, cancer, neurodegenerative diseases, ISG15-deficient inflammatory diseases

## Abstract

Type I Interferons (IFNs) induce the expression of >500 genes, which are collectively called ISGs (IFN-stimulated genes). One of the earliest ISGs induced by IFNs is *ISG15* (Interferon-Stimulated Gene 15). Free ISG15 protein synthesized from the *ISG15* gene is post-translationally conjugated to cellular proteins and is also secreted by cells into the extracellular milieu. ISG15 comprises two ubiquitin-like domains (UBL1 and UBL2), each of which bears a striking similarity to ubiquitin, accounting for its earlier name ubiquitin cross-reactive protein (UCRP). Like ubiquitin, ISG15 harbors a characteristic β-grasp fold in both UBL domains. UBL2 domain has a conserved C-terminal Gly-Gly motif through which cellular proteins are appended via an enzymatic cascade similar to ubiquitylation called ISGylation. ISG15 protein is minimally expressed under physiological conditions. However, its IFN-dependent expression is aberrantly elevated or compromised in various human diseases, including multiple types of cancer, neurodegenerative disorders (Ataxia Telangiectasia and Amyotrophic Lateral Sclerosis), inflammatory diseases (Mendelian Susceptibility to Mycobacterial Disease (MSMD), bacteriopathy and viropathy), and in the lumbar spinal cords of veterans exposed to Traumatic Brain Injury (TBI). ISG15 and ISGylation have both inhibitory and/or stimulatory roles in the etiology and pathogenesis of human diseases. Thus, ISG15 is considered a “double-edged sword” for human diseases in which its expression is elevated. Because of the roles of ISG15 and ISGylation in cancer cell proliferation, migration, and metastasis, conferring anti-cancer drug sensitivity to tumor cells, and its elevated expression in cancer, neurodegenerative disorders, and veterans exposed to TBI, both ISG15 and ISGylation are now considered diagnostic/prognostic biomarkers and therapeutic targets for these ailments. In the current review, we shall cover the exciting journey of ISG15, spanning three decades from the bench to the bedside.

## 1. Ubiquitin and Ubiquitylation

Ubiquitin is an 8.6 kDa ubiquitously-expressed eukaryotic regulatory protein first discovered by Dr. Gideon Goldstein in 1975 [1]. Drs. Irwin A. Rose, Avram Hershko, and Aaron Ciechanover further characterized the protein in the 1970s and 1980s, for which they won the 2004 Nobel Prize in Chemistry [2,3,4]. Ciechanover et al. initially observed ATP-dependent covalent attachment of a heat-stable polypeptide termed ATP-dependent proteolysis factor 1 (APF-1) to a lysozyme substrate [5]. The authors discovered multiple APF-1 molecules attached to a single substrate through isopeptide linkages and noted that these conjugated proteins degrade rapidly upon release of APF-1 [2,6]. In 1980, Drs. Keith Wilkinson, Michael Urban, and Arthur Haas showed that APF-1 described by Goldstein and Busch was ubiquitin and is ubiquitously expressed in most cells [7]. This early work on ubiquitin is summarized by Wilkinson et al. [4]. The structure of ubiquitin includes a β-grasp fold containing a four-strand antiparallel β-sheet surrounding a single α-helix [8]. The C-terminal glycine carboxyl group of ubiquitin was later recognized as its conjugation site to substrate lysine residues [9].

Ubiquitylation is the post-translational modification of a target substrate through the addition of one ubiquitin (monoubiquitylation) or a chain of ubiquitins (polyubiquitylation) [10,11,12,13]. Polyubiquitin chains are formed when an incoming ubiquitin conjugates to one of seven lysine residues on a previously appended ubiquitin [11,14]. Polyubiquitin chains with K48 linkages target substrates for proteasomal degradation [15]. K63 polyubiquitin linkages regulate endocytosis, cellular response to DNA damage, and cell signaling [16,17,18]. K6 linkages involve DNA repair regulation, K11 linkages play a role in ER (Endoplasmic Reticulum)-associated degradation, and K33 linkages facilitate kinase modifications [19,20,21]. Polyubiquitin chains can be assembled onto substrates by the addition of a thioester-activated ubiquitin to the distal K48 of a chain or by the proximal addition of K48 to the thioester-activated C terminus of a chain [22]. Recent studies from Ronchi et al. provide strong validation for the proximal indexation model [22].

Ubiquitylation occurs through a three-step enzymatic cascade [23]. In the initial step, ubiquitin is activated by an E1 ubiquitin-activating enzyme at the expense of ATP [24,25,26]. The E1 first binds to ATP and ubiquitin, while simultaneously catalyzing the acyl-adenylation of ubiquitin’s C-terminus, resulting in a ubiquitin-adenylate intermediate. Ubiquitin is then transferred to the E1 active site cysteine, creating a thioester linkage between the carboxyl group of ubiquitin’s C-terminus and the E1 active site cysteine’s sulfhydryl group [23,27,28]; AMP is then released. In the second step, the E2 ubiquitin-conjugating enzyme binds to both activated ubiquitin and the E1 ubiquitin-activating enzyme, transferring the ubiquitin from the E1 to its active site cysteine via transthiolation. The final step is catalyzed by E3 ubiquitin ligases, which form an isopeptide bond between a target substrate lysine and the C-terminal glycine of ubiquitin. Each E3 ligase can contain a homologous to the E6AP carboxyl terminus (HECT) domain or a Really Interesting New Gene (RING) domain, among several others [29]. HECT domains bind ubiquitin transiently and form a thioester intermediate with the E3 active site cysteine, whereas RING domains directly transfer ubiquitin from the E2 conjugating enzyme to its intended substrate [29]. This cascade is tightly regulated due to the hierarchical nature of the mechanism—one E1 enzyme binds to many E2s, which bind to hundreds of E3s [10]. For historical information on the ubiquitin conjugation system, we refer the readers to the original articles [23,24,25,26].

Canonically, polyubiquitin chains were thought to be formed only by the conjugation of the ubiquitin carboxyl-terminal glycine to an internal lysine residue of another ubiquitin [14]. However, a new type of chain, the linear polyubiquitin chain assembled on proteins by a novel ubiquitin ligase complex called LUBAC (linear ubiquitin chain-assembly complex), has been identified [30]. In these linear polyubiquitin chains, the C-terminal glycine of ubiquitin is conjugated to the α-amino group of the amino-terminal methionine of another ubiquitin. Although the tertiary structure of the linear chains is similar to that of K63 chains, recent findings have revealed that linear chain assembly requires a distinct ubiquitin-binding motif [31,32]. Interestingly, mutations in the domain specifically required for linear di-ubiquitin recognition of NEMO (NF-κB essential modulator) markedly reduce TNF-α-induced NF-κB activation [32]. Moreover, XR-MSMD (X-linked recessive Mendelian Susceptibility to Mycobacterial Diseases) is caused by hypomorphic mutations of NEMO [33], and mutations found in XR-MSMD (E315A and R319Q) have also been shown to be crucial for ubiquitin binding by UBAN (Ubiquitin-binding in NEMO and ABIN) [32]. Therefore, defects in linear polyubiquitination and the linear polyubiquitin-binding activity of NEMO are proposed to be an underlying cause of XR-MSMD diseases. Notably, mutations in ISG15 have been identified among other MSMD-causing mutations [34]. Recently, Fan et al. have demonstrated the existence of ISG15/Ub hybrid chains on proteins [35], and Juncker et al. have demonstrated their involvement in deregulating mitophagy. Whether mutated ISG15 forms Ub/ISG15 hybrid linear polyubiquitin chains and disrupts their function in MSMD is not known but is an interesting idea to explore.

Ubiquitylation is reversible. Deubiquitinases (DUBs) are proteases that cleave the peptide bond between ubiquitin and its substrate, reversing the cellular effects of ubiquitylation of that specific protein [36,37]. Deubiquitinases can remove both monoubiquitin and polyubiquitin chains, resulting in a single active unit of ubiquitin that can be recycled for further use. Approximately 100 DUBs have been identified in humans. They are categorized into seven families based on their structures/domains: the ubiquitin-specific proteases (USPs), the ovarian tumor proteases (OTUs), the JAB1/MPN/MOV34 metalloproteases (JAMMs), the ubiquitin C-terminal hydrolases (UCHs), the Josephins, the motif interacting with ubiquitin (MIU)-containing novel DUB family (MINDYs), and ZUP1 [38]. All DUBs, except for JAMMs, are classified as cysteine proteases.

The ubiquitin pathway regulates cell homeostasis by promoting the timely degradation of key proteins involved in regulating various vital cellular processes such as cell cycle, cell death, DNA replication and repair, transcription, and immune regulation, among numerous others [39,40,41,42,43]. Given the crucial role of the ubiquitin/26S proteasome pathway in maintaining cell homeostasis, it is not surprising that defects in the ubiquitin pathway lead to several pathological ailments such as cancer and neurodegenerative diseases, which are present leading causes of death worldwide.

## 2. Ubiquitin-Like Proteins

It is becoming increasingly clear that cellular processes are not exclusively regulated by ubiquitin but frequently require collateral signaling by other proteins called ubiquitin-like proteins [44,45,46]. Ubiquitin-like proteins are a family of small proteins structurally similar to ubiquitin, i.e., they also contain a β-grasp protein fold and a conserved C-terminal Gly-Gly motif [45,46]. Many ubiquitin-like proteins such as Small ubiquitin-like modifier (SUMO), Neural precursor cell-expressed developmentally down-regulated gene 8 (NEDD8, also known as Rub1), Autophagy-related protein 8 (ATG8), Autophagy-related protein 12 (ATG12), Ubiquitin-related modifier 1 (URM1), Ubiquitin-fold modifier 1 (UFM1), and HLA-F-adjacent transcript 10 (FAT10) have been identified and are known to post-translationally modify cellular proteins. However, in 1987 the first ubiquitin-like protein demonstrated to covalently modify proteins was ISG15 [47,48], the subject of the current review. In Table 1, we provide information on the precursor processing and cellular conjugation of some relatively well-studied Ubls and ISG15, and literature articles that provide detailed information on their structure and functions. We also refer readers to the selected reviews for more information on Ubls [45,49,50,51,52,53,54]. In Table 2, we provide landmark findings on ISG15, since it was discovered in 1979.

## 3. ISG15 and ISGylation

**Figure 1:**
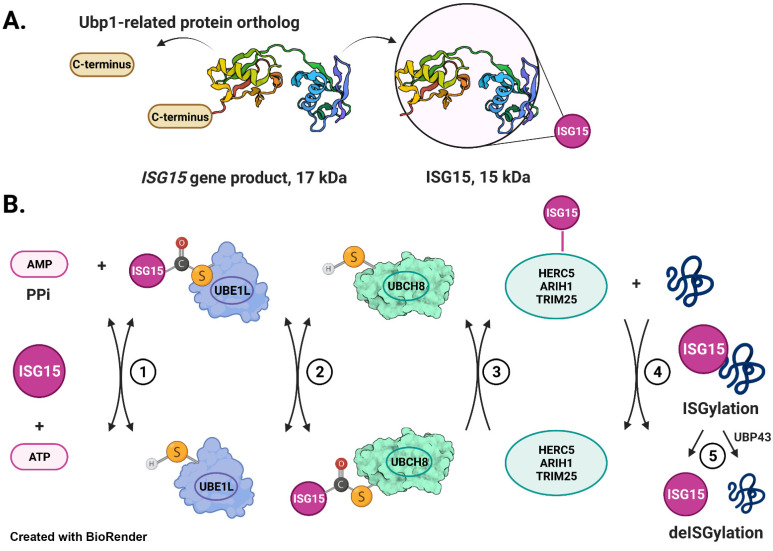
Processing of ISG15 and mechanism of ISG15 conjugation. (**A**) The schematic depicts that a 17 kDa precursor (Pro-ISG15) contains two ubiquitin-like domains joined by a flexible polypeptide hinge [69]. An ISG15-specific isopeptidase Ubp1-related protein exposes the C-terminal Gly-Gly motif necessary for conjugation to cellular proteins [87]. (**B**) E1 activating enzyme (Uba7/UBE1L) forms a high-energy thioester intermediate with ISG15 in an ATP-dependent manner (Step 1) [67,91]. E1 catalyzes the transfer of ISG15 thioester intermediate to the ISG15-specific conjugating enzyme E2 (UBCH8) to form UBCH8~ISG15 thioester (Step 2) [68]. ISG15-specific E3 ligase (HERC5, TRIM25, or ARIH1) binds the UBCH8~ISG15 thioester (Step 3) and catalyzes the aminolytic cleavage of UBCH8~ISG15 followed by the formation of an isopeptide bond with ε-NH2 of the Lys residue of the target protein (Step 4) [66,92,95]. UBP43 (USP18) catalyzes the process of deISGylation (Step 5) [88].

Interferon-Stimulated Gene 15 protein (ISG15 also known as UCRP, G1P2, IP17, IMD38, IFI15, IMD38) is expressed at low levels under physiological conditions in normal cells and tissues [70]. However, as the name indicates, its expression is strongly induced by type I interferons (IFN-α and β) through the binding of IFN regulatory factors (IRFs) to interferon-stimulated response element (ISRE)-containing promoters [86]. ISG15 was first identified as a 15 kDa protein in IFN-treated Ehrlich ascites tumor cells [59]. This 15 kDa protein was later found to be a mature form of a 17 kDa precursor protein (Pro-ISG15) (Figure 1A) [87]. Pro-ISG15 is comprised of two ubiquitin-like domains with marked homology to ubiquitin, accounting for its cross-reactivity to anti-ubiquitin antibodies and its earlier name ubiquitin cross-reactive protein (UCRP) [65]. A flexible polypeptide hinge joins the two ubiquitin-like domains (UBL1 and UBL2) in pro-ISG15, a conformation that is also retained in the 15 kDa mature form of ISG15. The processing of recombinant pro-ISG15 by a constitutive 100 kDa enzyme (not induced by type 1 IFNs) was demonstrated to expose the C-terminal double glycine residues (Gly-Gly) necessary for its subsequent conjugation to cellular proteins in a process called ISGylation. This 100 kDa processing enzyme was purified to apparent homogeneity and partial sequencing of a trypsin-derived peptide of the same indicated that this enzyme is either the human ortholog of yeast ubiquitin-specific enzyme (Ubp1) or a Ubp1-related protein [87]. As yeast do not contain ISG15, the authors suggested that this Ubp1 enzyme was recruited for pro-ISG15 processing in humans by adaptive divergence [87]. In 2012, using artificial substrates in which a cleavable extension is fused in the same way as in endogenous pro-ISG15, Malakhov et al. demonstrated that Ubiquitin-specific peptidase 18 (USP18, also known as UBP43) is capable of pro-ISG15 processing in vivo [88]. However, ISG15 conjugates are accumulated in *Ubp43^−/−^* mice and cells, suggesting that pro-ISG15 is being efficiently processed and the resultant ISG15 conjugated in the absence of UBP43. These results therefore argued against the role of UBP43 in the maturation of pro-ISG15. Thus far, the theory still holds true that Ubp1 (or Ubp1-related) enzyme may be a processing enzyme for pro-ISG15 in vivo.

Compared with ubiquitin, which is highly conserved (close to 100% identity), ISG15 shows substantial sequence variation from species to species. For example, mammalian and fish ISG15s share sequence identities of only 30–35%, whereas human and mouse ISG15 share only 66% sequence identity [89]. However, the C-terminal Gly-Gly required for ISGylation is conserved in ISG15s from all species.

ISGylation occurs through an enzymatic cascade similar to ubiquitin [90] but through enzymes distinct from those in ubiquitylation [48,91]. The E1 activating enzyme for ISG15, UBA7/UBE1L, activates the C-terminal group of ISG15 by forming a high-energy thioester intermediate in an ATP-dependent manner and transfers activated ISG15 to its E2 conjugating enzyme UBCH8 (Figure 1B, steps 1–2) [67,68]. UBCH8 then transfers ISG15 to one of the known E3 ligases such as HERC5 and 6 (HECT and RLD domain containing E3 ubiquitin protein ligases 5 and 6), and EFP (estrogen-responsive finger protein (also known as TRIM25)) (Figure 1B, step 3) [66,92,93,94]. E3 ligases finally append ISG15 to their designated substrates (Figure 1B, step 4). Both ubiquitin-like domains of ISG15 are required for conjugation to a substrate [95]. The ubiquitin-like 1 (UBL1) domain is necessary for the E3-mediated transfer of ISG15 from the E2 conjugating enzyme to the targeted substrate, whereas the ubiquitin-like 2 (UBL2) domain is required for ISG15 linkage to E1 activating and E2 conjugating enzymes. Two frameshift mutations (c.285del and c.299_312del, NM_005101.4 GRCh37(hg19) in the *ISG15* gene have been reported, which disrupt the UBL2 domain, resulting in the loss of the C-terminal Gly-Gly motif required for the function of the entire ISG15 protein in human subjects [82,83,84,96]. These mutations result in clinical manifestations such as recurrent ulcerative skin lesions, cerebral calcifications, and lung disease in afflicted human subjects [82,83,84]. These observations further affirm the importance of the UBL domain and C-terminus Gly-Gly motif for protein ISGylation and its subsequent biological effects on cellular/body functions in humans.

Similar to ubiquitylation, ISGylation is reversible. USP18 is the major ISG15-specific protease that cleaves ISG15-peptide linkages to remove ISG15 from conjugated proteins (Figure 1B, step 5) [88]. Several other ubiquitin-specific proteases (USPs) were subsequently found to remove conjugated ISG15 from their substrates [97]. For example, Ub-specific protease USP21 was found to cleave endogenous ISG15-modified substrates in IFN-treated cells and synthetic ISG15-AMC substrates [98].

ISGs encode all ISG15 conjugating and de-conjugating enzymes, i.e., their expression is regulated by interferons [99,100,101]. The enzymes of the ISG15 pathway are also upregulated upon lipopolysaccharide [102] and retinoic acid [103] treatments, and exposure to DNA damage or genotoxic reagents [104]. Both free and conjugated ISG15 pools are significantly induced upon treatment with type I IFNs. However, Loeb and Haas noted that free ISG15 increases early (within 30 min) during the IFN response and then undergoes conjugation (ISGylation) from 12 to 72 h [48]. Authors suggested that ISG-encoded ISG15 conjugating enzymes may not be efficiently induced (within 30 min) in all cell types after IFN treatment, consequently delaying ISGylation [48].

**Figure 2:**
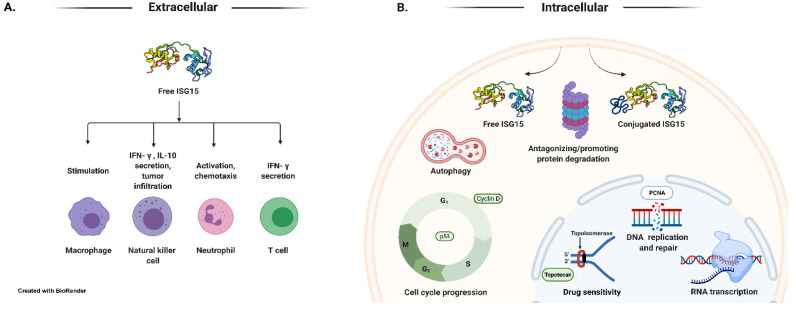
Functions of free and conjugated ISG15. (**A**) Extracellular free ISG15 serves as an immunomodulatory cytokine secreted in response to type I interferon induction [63,64]. ISG15 stimulates the production of IFN-γ in bovine peripheral blood mononuclear cells [60,106], T lymphocytes, and CD3^+^ T cells [63,107]. ISG15 stimulates the proliferation of CD56^+^ natural killer cells in the presence of CD3^+^ T cells, which leads to an enhanced lymphokine-activated non-major histocompatibility complex-restricted target cell lysis [63]. The binding of ISG15 to the LFA1 receptor on NK cells stimulates the release of IFN-γ and IL-10 from IL-12 primed NK cells [80]. Secreted ISG15 can also influence neutrophil chemotaxis [108]. (**B**) Free ISG15 protein synthesized from the *ISG15* gene is found in cells in its free and conjugated intracellular forms. Intracellular free ISG15 stabilizes (e.g., USP18) or destabilizes (e.g., Cyclin D1) some cellular proteins [72,109]. Intracellular ISG15 conjugates to a multitude of targets within the cell upon interferon stimulation. These protein targets are involved in every facet of cellular function, including DNA replication/repair, metabolism, signal transduction, and cytoskeletal organization, among several others [73,110,111,112,113]. The majority of the nuclear targets for ISG15 are involved in chromatin remodeling/RNA polymerase II transcription or RNA processing [114]. Protein ISGylation inhibits proteasome-mediated protein degradation and increases protein degradation by selective autophagy [70,76,115,116,117]. ISGylation confers topoisomerase-targeted drug sensitivity to tumor cells [74]. The consequences of ISGylation for ubiquitin-mediated protein degradation of substrates are variable and may be context-specific.

In addition to the intracellular pool of ISG15 conjugates, a large amount of free ISG15 is also detected inside the cells treated with IFNs. Intracellular free ISG15 is then conjugated to cellular proteins. Intracellular free ISG15 is also excreted in the extracellular milieu from IFN-treated cells, primary cultures of peripheral blood (including both CD4^+^ and CD8^+^ subpopulations), and viable cell lines of monocytes, T lymphocytes, B lymphocytes, and cells of epithelial origin (Figure 2A) [63,64,78,105]. Free ISG15 is also detectable in serum from healthy human volunteers treated with IFN-β [64].

## 4. Functions of Free ISG15 and ISGylation

In 2005, two proteomic studies were undertaken to help clarify the functions and the scope of ISG15 conjugation [114,118]. These studies revealed that ISG15 is conjugated to a multitude of targets within the cell upon interferon stimulation [114,118]. These cellular targets are involved in every aspect of cellular function, including DNA replication/repair, cell metabolism, signal transduction, and cytoskeletal organization, among several others [114,118]. One study found that most of the nuclear targets for ISG15 are involved in chromatin remodeling/RNA polymerase II transcription or RNA processing (Figure 2B) [114]. The consequence of ISG15 conjugation to the majority of other cellular targets remains unclear. On the other hand, in recent years, the development of genetic tools (siRNAs, shRNAs, and *Isg15*-, *Ubel1l*-, and *Ubp43*-KO mice [119,120,121,122] and in vitro enzyme assays [123], as well as the commercial availability of purified proteins/enzymes, have facilitated our understanding of the biological/cellular functions of ISG15.

Free ISG15 protein synthesized from the *ISG15* gene is found in cells in unconjugated and conjugated intracellular forms and is secreted extracellularly in its free form. Initial studies suggest that free ISG15 serves as an immunomodulatory cytokine secreted by a non-canonical mechanism in response to type I interferon induction [63]. It was demonstrated that ISG15 could stimulate production of IFN-γ in bovine peripheral blood mononuclear cells [106], T lymphocytes [63], and CD3^+^ T cells [107]. In addition, ISG15 stimulated the proliferation of CD56^+^ natural killer cells in the presence of CD3^+^ T cells, which resulted in an enhanced lymphokine-activated non-major histocompatibility complex-restricted target cell lysis [63]. Secreted ISG15 can also influence neutrophil chemotaxis [108]. More recent studies report the binding of ISG15 to lymphocyte function-associated antigen 1 (LFA1; also known as αLβ2 integrin, or CD11a/CD18), a receptor for extracellular ISG15 on NK cells. The binding of ISG15 to LFA1 stimulated the release of IFN-γ and interleukin-10 (IL-10) from IL-12 primed NK cells, and led to the activation of SRC family kinases (SFKs) [80]. Inhibition of SFKs can block cytokine secretion [80]. These findings further support a role for extracellular free ISG15 in cytokine signaling, which was initially reported by D’Cunha et al. [63,64].

Contrasting this cytokine-like activity, intracellular free ISG15 binds to non-structural protein 1 (NS1) of influenza A, resulting in viral replication inhibition [124,125]. In humans, intracellular free ISG15 binds to USP18, increasing its stability and leading to a decrease in IFN-α/β signaling. Therefore, patients lacking intracellular free ISG15, and consequently having low USP18 levels, display higher steady-state levels of ISG transcripts in whole blood than controls [126] and show greater resistance to viral challenge. Interestingly, Speer et al. have demonstrated that ISG15-mediated USP18 stabilization occurs in humans but not mice, and mice can regulate the IFN response in the absence of ISG15. Authors suggested that the novel ISG15-USP18 IFN regulation mechanism, which does not exist in mice, is most likely an evolutionary necessity for dampening the damaging effects of IFN (cytokine storm) in humans [126].

Interestingly, recent studies by Schwartzenburg et al. have revealed that protein ISGylation and MX-1 (Myxovirus resistance protein-1) protein levels, both induced by type I IFN, are increased in the peripheral blood mononuclear cells (PBMCs) from symptomatic (SARS-CoV-2-positive with symptoms) but not in asymptomatic patients (SARS-CoV-2-positive with no symptoms) and uninfected individuals (SARS-CoV-2-negative) [85]. It is not clear if increased levels of ISGylation and MX-1 are due the persistent elevation of IFN induction. Further studies are needed to determine if the sustained levels of ISGylation and MX1 are due to the lack of ISG15-mediated stabilization of USP18 in COVID-19 symptomatic patients [85].

Whereas ISG15 shares similar structural and mechanistic features with ubiquitin, the biological consequences of their conjugation (ISGylation vs. ubiquitylation) to cellular proteins are distinct. Ubiquitylation (Lys 48-linkage) targets proteins for degradation. On the other hand, there is extensive evidence first demonstrated by Desai et al. [70,76] that elevated ISGylation antagonizes the ubiquitin pathway by inhibiting polyubiquitylation and that this constitutive elevation of ISGylation mediates the mechanisms underlying disease pathogenesis [70,76]. A possible mechanism for broader inhibition of ubiquitin-dependent targeting pathways has been suggested by the observation that UBCH8~ISG15 acts as an alternate cognate for ligases specific to the closely related E2 conjugating enzymes: the ubiquitin-specific UBCH7 and UBCH5 clade [127]. Several groups have now demonstrated that ISG15 inhibits polyubiquitylation by modulating the activities of selected ubiquitin E2 and E3 ligases [128,129,130,131,132] and subsequent degradation of ubiquitin substrates. Recently, it has been reported that ISG15-ubiquitin mixed chains are formed on cellular proteins. These hybrid chains do not serve as a degradation/autophagy signal, negatively regulating the cellular turnover of ubiquitylated proteins [35]. Knowing that ubiquitin/ISG15 dual-function E3 ligases are elevated and able to conjugate both ubiquitin and ISG15 to cellular proteins in interferon-treated cells [92], it is reasonable to assume that the E3 ubiquitin ligase(s), which normally build polyubiquitin chains, conjugates both ISG15 and ubiquitin, producing atypical ubiquitin/ISG15 hybrid chains on proteins in cells overexpressing the ISG15 pathway. These hybrid chains are non-functional and do not transmit signals to proteasome/autophagy mediators (e.g., UBQL1, p62, etc.). Consequently, the degradation of these proteins may be inhibited in cells overexpressing ISG15.

In summary, ISG15 modulation of ubiquitin ligase activity and the formation of ISG15-ubiquitin mixed chains on substrates suggest that ISG15 inhibits protein degradation by altering the function(s) of E3 ubiquitin ligases. These observations further support the previously proposed model that ISG15 modulates polyubiquitylation and antagonizes the ubiquitin pathway [70].

Contrasting these results, some literature reports suggest that ISGylation can promote ubiquitin-dependent protein degradation by enhancing the enzymatic activities of ubiquitin E3 ligases or by conjugating to substrates. For example, HERC5-mediated ISGylation in the RING-in-between-RING domain of Parkin promotes Parkin’s ubiquitin E3 ligase activity [133]. Similarly, ISGylation of the carboxyl terminus of Hsp70-interacting protein (CHIP) promotes its ubiquitin ligase activity and inhibits lung cancer growth in response to type I IFN [134]. In addition, ISGylation triggers co-localization of multivesicular bodies (MVB) with lysosomes and promotes the aggregation and degradation of MVB proteins [135]. ISG15-dependent degradation of p53 represents an alternative mechanism of controlling the activity of p53 protein [110]. UBE1L induced ISGylation of the PML domain of PML/RARα causes its repression [73]. ISGylation of retinoic acid-inducible gene-I (RIG-I) decreases RIG-I cellular levels and downregulates RIG-I-mediated signaling [111]. Protein ISGylation increases protein degradation by selective autophagy [115]. ISGylation marks proteins for interaction with HDAC6 and p62 upon forced stressful conditions for their autophagic clearance [115]. ISGylation regulates essential mitochondrial processes including respiration and mitophagy, and influences macrophage innate immune signaling [116]. These results suggest that ISGylation is necessary for regulating mitochondrial functions and turnover. On the other hand, in the absence of ISG15 and ISGylation, the removal of damaged and dysfunctional mitochondria through mitophagy, a specialized form of autophagy involving the selective degradation and recycling of mitochondria, is reduced in IFN-treated *ISG15^−/−^* cells [117]. The constitutive elevation of the ISG15 pathway leads to defects in mitophagy and increases oxidative stress in A-T cells [117]. These two observations suggest that ISGylation dysregulates mitochondrial functions and turnover. See Figure 2B schematic for the known functions of intracellular free ISG15 and ISGylation, and extracellular secreted free ISG15. Additional studies have revealed that ISG15 conjugation to Serpin 2a, JAK, or STAT1 does not increase their respective rates of degradation [112,113]. The interferon-induced MxA protein is also a target of ISGylation. However, functional consequences of MxA-ISGylation are not clear [114].

Taken together, these empirical findings suggest that the consequences of elevated ISGylation on ubiquitin-mediated protein degradation of substrates are variable and could be context/stress stimuli-specific or experimental condition-specific. Considering the contribution of the ISG15 pathway to the etiology of viropathy, malignancy, and neurodegenerative disorders, further studies are urgently needed to elucidate the functions of ISG15 and ISGylation in human disease.

## 5. The ISG15 Pathway in Human Diseases

ISG15 is minimally expressed under normal physiological conditions. However, the constitutive elevation of ISG15 is noted in cancer [70], neurodegenerative diseases [76], upon TBI [81], and in response to pathogen infections [78,136]. Several mechanisms have been demonstrated through which free ISG15 and ISGylation possibly contribute to the etiology of human ailments. ISG15 is involved through marking proteins for degradation, antagonizing protein degradation (stabilizing proteins), affecting protein localization, preventing the formation of protein complexes, or regulating immune response(s) [96,126,136,137,138,139,140,141,142,143,144,145,146,147,148,149,150]. In the current review, we shall focus on the role of the ISG15 pathway in human diseases through protein degradation.

### 5.1. ISG15 in Cancer

“When protein destruction runs amok, malignancy is on the loose” [151]. Supportive of the quote, defects in ubiquitin-mediated protein degradation have been identified as a hallmark of cancer. An emerging body of evidence reveals that the ISG15 pathway, an antagonist of the ubiquitin pathway, is aberrantly elevated in various human malignancies, implicating its potential role underlying the observed defects in protein degradation in cancer. Concurrently, it has been reported that the ISG15 pathway (ISG15 and its conjugating enzymes) is overexpressed in human breast cancer cells and clinical specimens [70,71,152,153] and inhibits protein degradation in cancer cells [70]. Specifically, elevated ISGylation inhibits the camptothecin-dependent proteasome-mediated degradation of topoisomerase I in breast cancer cells, accounting for camptothecin’s efficacy as an anticancer chemotherapeutic [70]. The inhibition of topoisomerase I degradation by ISG15, in turn, confers camptothecin sensitivity to tumor cells [70]. These findings led to the hypothesis that ISGylation could serve as a tumor biomarker for camptothecin sensitivity/resistance in cancer patients, recently confirmed in several independent clinical trials conducted by other groups in the United States and China [154,155,156]. This was the first proof-of-principle study that demonstrated the function of ISG15 in antagonizing the ubiquitin pathway [74].

In parallel studies, similar to topoisomerase I, constitutively elevated ISGylation inhibits cellular protein polyubiquitylation and protein turnover in tumor cells [70]. Using a gene silencing approach, ISGylation blocks proteasome-dependent degradation of protumor proteins (e.g., NFAT5, S100A4) and promotes breast cell transformation [79,157]. Based on these observations, Desai et al. concluded that ISG15 promotes tumorigenesis by inhibiting ubiquitin-mediated degradation, consequently stabilizing oncoproteins. Consistent with this conclusion, ISG15 and UBCH8 (ISG15-specific conjugating enzyme) promote breast cancer cell migration by disrupting F-actin architecture and the formation of focal adhesions [70]. A subsequent study demonstrated the role of the Ki-Ras/ISG15-axis in the malignant transformation of breast cancer cells, i.e., oncogenic Ki-Ras regulates the expression of the ISG15 pathway, and ISG15, in turn, stabilizes Ki-Ras protein by inhibiting its targeted degradation via lysosomes in breast cancer cells [157]. Inhibition of this loop by silencing either Ki-Ras or the ISG15 pathway restored the disrupted cellular architecture of breast cancer cells, which is a hallmark of most cancer cells [157]. Likewise, Hermann et al. identified ISG15 as an integrin-induced MRTF–SRF target gene using a breast cancer model [158]. This group reported that high levels of fibronectin-binding integrins and ISG15 promote the invasion of the malignant MDA-MB-231 breast cancer cells and correlate with poor survival rates in a large cohort of breast cancer patients [158]. Another group reported that ISGylation drives basal breast tumor progression by promoting EGFR recycling and Akt signaling [159]. Additionally, in breast cancer, high mRNA and protein ISG15 expression are associated with lymphovascular invasion (LVI), higher histological grade, larger tumor size, hormonal receptor negativity, HER2 positivity, HER2-enriched breast cancer subtypes, immune markers (CD8, FOXP3, and CD68), and with poor patient outcome [152]. Together, these results led to the general notion that aberrant activation of the ISG15 pathway may be conferring a motile phenotype to breast cancer cells by disrupting cell architecture and stabilizing proteins involved in breast cancer cell migration, invasion, and promoting breast tumorigenesis by stabilizing oncoproteins [79,157].

Because the cellular architecture is conserved and the ISG15 pathway is constitutively activated in a variety of tumors [92,160,161], it is reasonable to assume that the breast cancer observations must hold true for many other tumors. As expected, subsequent studies from distinct groups have revealed that the ISG15 pathway knockdown reverses the Ki-Ras-associated phenotypes such as increased proliferation and colony formation of pancreatic ductal adenocarcinoma (PDAC) cells [162]. Moreover, ISG15 promoted malignant phenotypes of esophageal squamous cells, including proliferation, migration, invasion, and tumor formation in vivo [163]. Furthermore, ISG15 promotes the proliferation and migration of hepatocarcinoma (HCC) cells through maintaining Survivin protein stabilization via sequestering XIAP from interacting with Survivin [164]. Knocking down ISG15 with small interfering RNA (siRNA) inhibited xenografted HCC tumor growth and prolonged the lifespan of tumor-bearing mice [164]. More recently, Lo et al. reported that endothelial lipase (LIPG) executes its oncogenic function through its involvement in interferon-related DTX3L-ISG15 signaling. DTX3L, an E3-ubiquitin ligase, is required for maintaining LIPG protein levels by inhibiting proteasome-mediated LIPG degradation, and DTX3L-LIPG-ISG15 signaling is essential for malignancies of triple-negative breast cancer cells (TNBCs) [165]. ISG15 has been identified as a critical microenvironmental factor for pancreatic cancer stem cells [166]. Inhibition of ISG15/ISGylation impaired the self-renewal and tumorigenic potential of pancreatic cancer stem cells [167]. ISG15 is expressed on nasopharyngeal carcinoma (NPC) cells and is related to a poor prognosis of patients with NPC [168]. Tumor cell-secreted ISG15, which acts as a tumor microenvironmental factor, induces an M2-like phenotype, promoting tumor progression and suppression of cytotoxic T lymphocyte response [168]. Together, these results suggest that constitutively elevated expression of ISG15 and its conjugates are intrinsic features of human malignancies that trigger tumorigenesis and metastasis.

Contrasting these observations that ISGylation promotes tumorigenesis by stabilizing oncoproteins, several studies have revealed that ISGylation inhibits tumorigenesis by destabilizing growth regulatory proteins [169]. For example, UBE1L targets cyclin D1 [72] and PML-RARα [73] for proteasomal degradation, suppressing the growth of human lung cancer cells. In subsequent studies, engineered repression of USP18/UBP43 (ISG15 deconjugating enzyme) was shown to destabilize PML-RARα, reduce proliferation, and increase apoptosis in acute promyelocytic leukemia and lung cancer cell lines [169,170,171]. Likewise, genetic loss of USP18 repressed cancer formation in engineered murine lung cancer models [169]. Moreover, USP18 knockdown destabilizes PTEN, whereas USP18 overexpression stabilized PTEN protein [172]. These results suggest that ISGylation serves as a degradation signal and promotes proteasomal degradation of cyclin D1, PML-RARα, and PTEN in lung cancer cells. Notably, both UBE1L and UBCH8 knock-down cells show decreased ISGylation and increased levels of free ISG15 [75,157,173]. As expected, incubation with purified USP18 decreased ISG15 conjugates and increased levels of free ISG15 [88]. Therefore, in all three cases (*UBCH8^−/−^*, *UBE1L^−/−^*, and incubation with USP18), free ISG15 is increased irrespective of cell type and experimental models used in these studies. Notably, UBE1L is undetectable [174], and USP18 expression is higher [170] in cancer vs. normal cells/tissues. Intracellular levels of free ISG15 are expected to increase due to decreased ISGylation (due to low levels of UBE1L) and increased ISG15 deconjugation (due to high levels of USP18) of cellular proteins in these cancer tissues. Interestingly, it has been reported that free ISG15 stabilizes USP18 by preventing SKP2-dependent ubiquitination, and SKP2 has been implicated in Cyclin D1 degradation [109]. It is possible that accumulated free ISG15 stabilizes USP18 in cancer cells expressing low levels of UBE1L and high levels USP18, which in turn removes the ISG15 degradation signal from the target proteins (e.g., Cyclin D1), consequently stabilizing these protumor proteins. Such studies are warranted as free ISG15, which has been identified as an antineoplastic agent and immune modulator.

It has been demonstrated that free ISG15 inhibits tumor growth when added extracellularly and induces the infiltration of NK cells in tumors grown in nude mice, and intracellular free ISG15 enhances 26S proteasome-dependent surface expression of MHC Class I complexes on breast cancer cells [173]. These results suggest that free ISG15 exerts an antitumor effect by activating the innate and adaptive arms of the immune system in vivo and support the conclusions of early in vitro cell culture studies that free ISG15 has an immunocytokine-like function. Strategies to increase systemic levels of free ISG15 by targeting UBE1L and UBCH8 may therefore be beneficial for cancer patients.

The microarray data from the Oncomine^TM^ cancer Profiling Database reveal that ISG15 gene expression is elevated in various human malignancies such as breast, colon, tongue, and ovarian, among numerous others, compared to their normal counterparts. In addition, stage-specific elevated expression of ISG15 in human solid tumors is documented in the same database and by Anderson et al. [175]. IFN-mediated ISG15 expression is regulated by NFkB [176], retinoic acid [103,177], and JNK [178] in tumor cells. Moreover, IFN-mediated ISG15 expression is governed by several tumor suppressors and oncogenes. For example, simian virus 40 T antigen (SV40 T-antigen) induces expression of ISG15 in HeLa [179], 293T [179], and WI38 human fibroblast cells [70,137]. Similar to SV40 T-antigen, ISG15 expression is also regulated by E1A oncoprotein, as well as tumor suppressors p53 [180,181], BRCA2 [182], and ATM [65,76]. IFN-mediated ISG15 expression is induced by certain genotoxic stresses [180]. On the other hand, p53 [181] and telomerase [183] induce the IFN-independent expression of ISG15. Thus, it is very clear that *ISG15* gene/protein expression is elevated in most cancers.

On the other hand, very little information is available on the expression levels of free ISG15 protein vs. its conjugates in human cancers. A small study has demonstrated that the expression of free ISG15 and ISG15 conjugates is heterogeneous in various human tumors [70]. Desai et al. found that some tumors express very high levels of ISGylation and low levels of free ISG15, while others display high levels of free ISG15 and low levels of ISGylation. The reasons for the heterogeneous expression of ISG15 conjugates in tumors are not known. However, Old World Monkey ISG15 (OWmISG15) more efficiently ISGylates proteins compared to human ISG15 in human, monkey, and mouse cell lines [184]. The introduction of an N89D mutation in HuISG15 improved its ISGylation capacity, and additional Q31K/T33A/D133N mutations yielded a human ISG15 variant with an ISGylation efficiency comparable to OWmISG15 [184]. Whether the heterogeneous expression of ISG15 conjugates is due to the differential expression of ISG15-conjugating/deconjugating enzymes or mutations in ISG15 protein in human cancers is not known. Moreover, whether high levels of free ISG15 and low levels of conjugates correlate with a better outcome for patients and vice versa is understudied. All available data suggest that intracellular ISG15 conjugates and free ISG15 could harm patients by stabilizing cellular proteins that promote cancer, and secreted free ISG15 may benefit patients by modulating immune system functions [185]. Thus, ISG15 clearly has double-edged functions in malignant cancers, and proper consideration must be given to assess risk-benefit prior to administering ISG15-targeted cancer therapy to cancer patients when available.

### 5.2. ISG15 in Neurodegenerative Disorders

ISG15 (UCRP) and LMP2 pools were found to be basally elevated in human fibroblasts with the inherited rare neurodegenerative disease Ataxia Telangiectasia (A-T) due to constitutive activation of the IFN-β induction pathway, first demonstrated by Siddoo-Atwal et al. [65]. More recent studies have revealed that ISG15 is indeed elevated in fibroblasts derived from A-T patients [65] and brain tissue from both A-T patients and *Atm* knockout mice [76]. Notably, elevated expression of ISG15 in A-T is three-fold higher in the cerebellum, a part of the brain affected in A-T, compared to cerebral tissue in the same mice [186]. Similar to cancer, defective protein degradation is a hallmark of neurodegenerative disorders [187]. Likewise, the 26S/proteasome-mediated degradation is impaired in fibroblasts derived from A-T patients, and this impairment is associated with elevated ISG15 expression [76]. Moreover, K63-linked polyubiquitin and ISG15 inclusions/aggregates are found in post-mortem brain tissues from A-T patients [76], a hallmark of defective proteasome function in other neurodegenerative disorders. There is also evidence that basal autophagy is upregulated to compensate for impaired ubiquitin-mediated degradation in A-T cells where ISG15 is elevated [188]. Genotoxins like UV radiation overactivate autophagy and induce abnormal autophagic degradation of substrates in A-T cells. Both basal and UV-activated autophagy are attenuated in A-T cells treated with ISG15 and UBCH8 short hairpin RNAs (shRNA), which simultaneously restores proteasome function [188]. More recently, Juncker et al. demonstrated that elevated ISG15 inhibits congression of abnormal mitochondria (mito-aggresomes) at the perinuclear region, consequently inhibiting their degradation via mitophagy in A-T cells [117]. This inhibition is evident by the restoration of mitochondrial turnover following the shRNA-mediated knockdown of free ISG15 in A-T cells [117]. These results suggest that aberrant activation of the ISG15 pathway leads to proteinopathy and mitochondriopathy in A-T cells.

Similar to A-T, the ISG15 pathway is aberrantly elevated in the spinal cords of human ALS patients and SOD1^G93A^ ALS mice, first demonstrated by Wang et al. [77]. Our group extended these observations, finding that ISGylation is elevated in the lumbar spinal cords, cerebrospinal fluid, and cultured lymphocyte cell lines obtained postmortem from ALS patients [81]. Strikingly, the United States Department of Veterans Affairs (VA) and the Department of Defense (DoD) has revealed that veterans who have served in the military are at a nearly 60% greater risk of being diagnosed with ALS than those with no history of military service [189,190,191]. TBI has been identified as one of the major risk factors for ALS development in veterans and civilians [192,193]. However, the causes underlying ALS development and whether ALS development is due to the TBI exposures during military service are not known. Schwartzenburg et al. noted that ISGylation is elevated in the lumbar spinal cords and CSFs obtained from TBI-exposed veterans who were later diagnosed with ALS (TBI-ALS) compared to levels in veterans with ALS with no history of TBI (non-TBI ALS) and healthy subjects (control) [81]. Levels of ISG15 conjugates did not change in occipital lobe samples from the same patients, suggesting elevated ISGylation to be distinct to the ALS disease-specific lumbar spinal cord [81]. Similar to A-T and other neurodegenerative disorders, defects in protein degradation are characteristic of ALS [194,195]. However, if elevated ISGylation inhibits protein degradation and leads to neurodegeneration in ALS and TBI-exposed veterans has not been studied to date and needs further investigation.

Parkin, an E3 ligase, has been identified as a novel target of ISG15 conjugation. ISG15 modification affects toxic protein accumulation by positively modulating Parkin activity [133]. Based on these observations, Im et al. suggested that ISG15 conjugation is important for maintaining neuronal cell viability, and its alteration could promote neurodegeneration in Parkinson’s Disease [133]. However, if ISG15 is elevated in Parkinson’s patients has not been studied. Elevated ISG15 is also seen during inflammation and neuronal injury from TBI. ISG15 is dramatically elevated in the brains of mice subjected to global ischemia and TBI, and in transgenic mice overexpressing HIV gp120 protein [196]. These results suggest that activation of ISGs is a shared feature of neuronal injuries and that ISG15 may be a suitable biomarker for detecting neuronal injuries in the central nervous system (CNS) [196]. Consistent with these findings, upregulation of ISG15 was observed in neuronal tissues and has been linked to degeneration of sensory neurons in *Clec16a* knockout mice [197]. Together, these results suggest that elevated ISG15 is associated with neurodegeneration. However, if it is causally related to neurodegeneration requires further investigation.

### 5.3. ISG15 in Viral and Bacterial Pathogenesis

In 1987, Haas et al. reported that ISG15 expression is elevated within one hour of viral infection in host cells and is correlated with resistance to viral infection in cell culture studies [47]. After two decades, Ketscher et al. reported that inhibition of USP18 deISGylation activity enhances ISGylation and viral resistance [198], supporting the earlier observations by Haas et al. Several groups have now endorsed an anti-viral function of ISG15 and ISGylation against murine Gammaherpesvirus, Influenza A and B viruses (IAV), Sindbis virus (SNV), Vaccinia virus, Herpes simplex virus 1 (HSV-1), Chikungunya virus, murine Norovirus, Ebola virus, Dengue virus, and West Nile virus in cell culture, animal, and human studies [96,126,136,142,146,147]. Whether ISG15/ISGylation plays a role in establishing anti-viral immunity against the SARS-CoV-2 virus is unknown; however, there are literature reports implicating the same [145,199,200].

Similar to cancer and neurodegenerative disease models, ISG15 and ISGylation exert antiviral responses by inhibiting or promoting protein degradation. For example, ISG15 inhibits the monoubiquitination of viral proteins essential for the budding of viruses (e.g., Ebola, among several other viruses) and consequently inhibits viral release [129]. ISGylation of Interferon Regulatory Factor 3 (IRF3) sustains its activation and enhances IRF3-mediated anti-viral responses by inhibiting its degradation [201]. ISGylation of NEDD4 prevents viral matrix protein VP40 ubiquitination and inhibits budding [128,129,202]. ISGylation inhibits Gag polyprotein ubiquitylation in HIV-1 and its host tumor susceptibility gene 101 protein (TSG101). This inhibition results in disruption of Gag and TSG101 interaction, preventing virion assembly and release from infected cells [203]: processes necessary for HIV-1 budding and release [204]. Contrasting these antagonizing effects, ISGylation of misfolded p53 by HERC5 and TRIM25 leads to p53 degradation, and ISG15 deficiency accumulates misfolded p53 and enhances HIV-1 replication [205]. ISG15 increases basal and infection-induced autophagy during *Listeria monocytogenes* infection by modifying mTOR, WIPI2, AMBRA1, and RAB7 [206].

In addition to these functions of ISGylation in protein degradation/stability, ISG15 disrupts viral functions through distinct mechanisms. For example, HERC5 conjugates ISG15 to the newly synthesized proteins in a co-translational manner. Newly translated viral proteins have been suggested to be the primary targets of ISG15 [207]. Consistent with this suggestion, ISGylation of human papillomavirus (HPV) L1 capsid protein has a dominant-inhibitory effect on the infectivity of HPV16 pseudoviruses [207]. ISG15 binds to NS1 protein of influenza A, resulting in viral replication inhibition [124,125]. Influenza virus nucleoprotein (NP) and matrix protein (M1) are ISGylated, which hinders the oligomerization of the more abundant unconjugated NP. Therefore, ISG15 acts as a dominant-negative inhibitor of NP oligomerization and impedes the formation of viral ribonucleoproteins, resulting in decreased viral protein synthesis and virus replication [208]. ISGylation of the HCMV (Human cytomegalovirus) scaffold protein pUL26 interferes with the viral modulation of the innate immune response [209]. It is possible that these antiviral roles of ISG15/ISGylation may be due to the stabilization/degradation of the cellular/viral proteins involved. However, that has not been suggested or tested in any of these reports.

Given the importance of the anti-viral response governed by ISG15, it is not surprising that viruses have evolved strategies to counteract their anti-viral effects. For example, influenza B virus NS1 protein, Vaccinia virus E3 protein, and HCMV IE1 and PUL26 proteins inhibit the anti-viral function of ISG15 by preventing ISGylation of host cellular proteins [126]. Additionally, HIV inactivates the IFN-α anti-viral response in infected patients [210]. The SARS coronavirus papain-like protease (SARS-CoV PLpro) exerts deubiquitination/deISGylation activity, and PLpro inhibitors protect mice from lethal infection by coronaviruses in vivo [211]. ISG15 conjugation is essential for antiviral IFN responses mediated by the viral RNA sensor MDA5. ISG15-dependent activation of MDA5 is antagonized through direct deISGylation mediated by the papain-like protease of SARS-CoV-2, suggesting a crucial role for ISG15 in the MDA5-mediated antiviral response [200]. These results make ISGylation in COVID-19 and PLpro inhibition promising treatment targets. Because ISG15 is elevated and has anti-viral roles, ISG15 has been identified as a diagnostic and molecular target for the treatment of patients with SARS-CoV-2 infection [212].

Contrasting these reports, ISG15 also has pro-viral functions. ISG15 has been identified as a pro-viral factor for the hepatitis C virus and a regulator of the IFN response [213,214]. A recent study by Bogunovic et al. has revealed that ISG15 also has an antibacterial function [78]. This study demonstrated that children carrying a genetic mutation in the *ISG15* gene were highly susceptible to intracellular bacterial infections [78]. Notably, the Bogunovic et al. study revealed that IFN-γ production was highly impaired in ISG15-deficient children, and this defect is within the NK cell population [78]. IFN-γ production is crucial to fighting intracellular bacterial infections [78], and purified free ISG15 was previously shown to activate human NK cells and induce secretion of IFN-γ in vitro [63]. Together, these two independent observations suggest that ISG15-mediated induction of IFN-γ is essential for the establishment of immunity against bacteria, a process likely mediated in part by NK cells. Influenza B virus NS1 protein counteracts ISG15 antiviral activity by sequestering ISGylated viral proteins [208].

### 5.4. ISG15/ISGylation in Human Inflammatory Diseases

ISG15 was found to be one of the nine Mendelian Susceptibilities to Mycobacterial Disease (MSMD)-causing genes [34]. The empirical evidence collected to date for six ISG15-deficient individuals indicates that the lack of free secreted ISG15 (extracellular ISG15) underlies increased susceptibility to mycobacterial infection (a primary clinical presentation) in these patients [78]. In contrast, the primary clinical presentation in a family from China with complete ISG15 deficiency was intermittent seizures stemming from intracranial calcifications. Lack of intracellular free ISG15 prevents the accumulation of USP18, a known negative regulator of IFN-α/β, resulting in enhanced IFN-α/β immunity and autoinflammation in the patients from China [78]. All patients showed peculiar skin lesions—phenotypes that are not frequently observed in patients with type I interferonopathy [78]. Systemic type I IFN inflammation due to ISG15 deficiency has been identified as a cause of skin lesions in ISG15 deficient patients [78,82,83,84]. The presence of high levels of pSTAT1 and CD68^+^pSTAT1^+^ macrophages within these lesions suggested increased inflammation and pathological skin features in keratinocytes and myeloid cells, respectively, in ISG15-deficient patients.

In addition to MSMD, an increasing number of genetic diseases belonging to type I interferonopathy have been discovered, which includes *ISG15* gene deficiency. A less severe form of Aicardi-Goutieres syndrome (AGS) is one of the type I interferonopathy diseases, which is caused by mutations in the *ISG15* gene [78,215]. In a distinct study, two novel compound heterozygous variants ((c.285del and c.299_312del, NM_005101.4 GRCh37 (hg19), discussed in Section 3) both classified as pathogenic according to ACMG criteria) in the *ISG15* gene, resulted in a complete ISG15 deficiency due to disruption of the second ubiquitin domain of the corresponding protein [82]. The clinical phenotypes of this patient include recurrent pulmonary manifestations, the absence of mycobacterial infections, and inflammatory skin lesions. The reasons for the phenotype seen in this patient are not known but may be due to the systemic type I IFN inflammation caused by ISG15 deficiency as described by Bogunovic et al. [78]. ISG15 deficiency can therefore be considered a bona fide type I interferonopathy caused by inflammation.

Very recently, ISG15 has been identified as a novel mediator of vascular damage in hypertension through oxidative stress and inflammation [216]. ISG15 strongly correlates with inflammation and disease severity during active tuberculosis (TB) [217]. ISG15 secretion exacerbates inflammation in SARS-CoV-2 infection [199]. ISG15-mediated inflammation leads to Maternal Immune Activation (MIA)-induced psychiatric disorders in offspring [218]. Autosomal recessive ISG15 deficiency causes type I interferonopathy with systemic lupus erythematosus (SLE) and inflammatory myositis [219]. Type I IFN also induces protein ISGylation to enhance cytokine expression and augments colonic inflammation [220]. Inflammation plays a critical role in accelerating the progression of neurodegenerative diseases, such as A-T [221] and ALS [222]. Inflammation is also a hallmark of human malignancies [223,224]. Moreover, TBI induces inflammation in the brain [225]. In all cases, ISG15 is elevated [70,76,81]. Together, these results suggest that ISG15 modulates IFN-induced inflammation and justify the use of elevated ISGylation as a potential biomarker for diagnosing these inflammatory diseases [70,76,81]. The known functions of ISG15 in human diseases described in this review are summarized in Table 3.

## 6. Conclusions

Inflammatory diseases (such as bacterial coinfections with SARS-CoV-2 virus), cancer, neurodegenerative disorders, and TBI are leading causes of death worldwide. Free ISG15 and its conjugates, which are barely detected in most normal tissues, are aberrantly elevated under these pathological ailments. It is apparent from literature studies that both free ISG15 and its conjugates are initially elevated as a protective mechanism against pathogens in pre-malignant cells and damaged neurons. However, this ISG15/ISGylation-mediated protective mechanism is dysregulated over time, causing damage to the cells, and leading to pathology.

Ample evidence suggests that aberrant ISG15/ISGylation may promote tumorigenesis, neurodegeneration, and inflammation. Understanding how the ISG15 pathway is deregulated and promotes viropathology, oncotransformation, and neurodegeneration may identify ISG15/ISGylation as potential target(s) for developing therapies for these fatal diseases that currently do not have a cure. Thus, ISG15, ISGylation, and ISG15 conjugating enzymes are emerging as potential biomarkers for the clinical diagnosis of diseases discussed in the current review. Furthermore, increased ISG15 conjugation confers anti-cancer drug (e.g., Topotecan) sensitivity to tumors, validating the potential of ISG15 conjugates to serve as a biomarker for drug sensitivity in cancer patients [74].

In summary, ISGylation is emerging as a key protein in strategies for diagnosis, prognosis, and therapies of cancer, neurodegenerative and inflammatory diseases, viropathy, and bacteriopathy. However, it is important to take note of empirical evidence suggesting that free ISG15 has the potential to boost the immune system, and ISGylation may be contributing to pathology. Further investigation on the functions of ISGylation/free ISG15 should be taken into consideration to assess risks and benefits prior to administering ISG15-targeted cancer therapy to patients when available.

## 7. Patents

1. Desai: S. Therapeutic and Diagnostic Method for Ataxia-Telangiectasia. US Patent 9,599,626, 21 March 2017.
2. Desai, S. (2021). Compositions and Methods for Detecting Proteinopathies. US Patent 10,962,553, 30 March 2021.

## Figures and Tables

**Table 1:** Ubiquitin-like proteins: processing and conjugation.

Ubl	Requires Processing of Premature Form for Conjugation	E1, E2, and E3s	Consensus Sequence Required for Conjugation	Chain Formation on Substrates	Conjugation Required for Function(s)	Orthologs in Yeast
**SUMO1-4**	Yes	**E1:** UBA2/SAE1 **E2:** UBC9 **E3:** RanBP2, PIAS1, PIAS3, PIASxα, PIASxβ and PIASy, PC2 (CBX4), HDAC4, TOPORS, RHES, TRIM(s) (reviewed in [55])	Yes	Yes	Yes	Yes
**NEDD8**	Yes	**E1:** UBA3/NAE1 **E2:** UBC12, UBE2F **E3:** RBX1, RBX2 (reviewed in [56])	No	Yes	Yes	Yes
**ATG8**	Yes	**E1:** ATG7 **E2:** ATG3 **E3:** ATG5-ATG12 [57]	No	No	Yes	Yes
**ATG12**	No	**E1:** ATG7 **E2:** ATG10	No	No	Yes	Yes
**URM1**	No	**E1:** UBA4	-	-	Yes	-
**UFM1**	Yes	**E1:** UBA5 **E2:** UFC1 [49]	No	-	Yes	-
**FAT10**	No	**E1:** UBA6 **E2:** UBE2Z **E3:** Parkin [58]	No	Yes	Yes	-
**ISG15**	Yes	**E1:** UBE1L/UBA7 **E2:** UBCH8 **E3:** HERC5, EFP	No	Not known	Yes/No *	No

* Intracellular free ISG15 interacts with USP18 (non-covalent interaction), increasing its stability and leading to a decrease in IFN-α/β signaling. Extracellular free ISG15 serves as an immunomodulatory cytokine secreted by a non-canonical mechanism in response to type-I interferon induction. Intracellular free ISG15 regulates cellular function(s) by conjugating (covalent conjugation) to the cellular proteins. Since this review focuses on ISG15, information associated with it is highlighted in a grey shade in this table.

**Table 2:** ISG15: journey from bench to bedside.

Year	Landmark Findings	References *
1979	ISG15 was identified as a 15 kDa protein in IFN-treated Ehrlich tumor cells	[59]
1984	ISG15 was detected in human and bovine cell lines treated with Type I IFNs	[60]
1987	ISG15 was identified as a Ubiquitin-Cross-Reactive Protein (UCRP)	[47]
1987	UCRP was renamed as ISG15	[61]
1987	ISG15 was found to be an antiviral protein	[47]
1988	A 15 kDa ISG15 protein was found to be a mature form of a 17 kDa precursor protein (Pro-ISG15)	[62]
1992	ISG15 was found to be conjugated to cellular proteins	[48]
1996	ISG15 was found to be an interferon-induced cytokine	[63,64]
1996	ISG15 and LMP2 were found to be constitutively elevated in response to ATM loss-of-function in Ataxia Telangiectasia	[65]
2001–2006	Enzymes mediating ISG15 conjugation were identified	[66,67,68]
2005	A three-dimensional structure of ISG15 was determined	[69]
2006	ISG15 is elevated and inhibits the canonical ubiquitin/26S proteasome pathway in breast cancer	[70]
2008	ISG15 was identified as a potential prognostic marker in human breast cancer	[71]
2008	ISG15 may be promoting protein degradation	[72,73]
2008	ISG15 was identified as a novel tumor biomarker for drug sensitivity	[74]
2009	ISG15 in innate immunity	[75]
2011	ISG15 is elevated and inhibits the canonical ubiquitin/26S proteasome pathway in Atm null Ataxia Telangiectasia cells.	[76]
2011	ISG15 was found to be elevated in Amyotrophic Lateral Sclerosis	[77]
2012	Inherited ISG15 deficiency was found to be associated with severe mycobacterial disease in humans	[78]
2014	ISG15 was found to be one of the nine Mendelian Susceptibilities to Mycobacterial Disease (MSMD)-causing genes	[34]
2013	ISG15 was found to disrupt cytoskeletal architecture and promote motility of human breast cancer cells (ISG15 promotes tumorigenesis).	[79]
2017	Lymphocyte function-associated antigen 1 receptor (LFA1) has recently been identified as the cellular receptor for ISG15	[80]
2019	ISGylation is increased in cases of TBI-Exposed ALS veterans	[81]
2020–2021	Inflammatory cutaneous lesions and pulmonary manifestations were noted in a patient with autosomal recessive ISG15 deficiency	[82,83,84]
2022	ISGylation is increased in the peripheral blood mononuclear cells derived from symptomatic COVID-19 patients	[85]

* Pioneer findings that are supported by similar findings from distinct groups (see references cited in this review).

**Table 3:** The ISG15 Pathway in human diseases.

Cancer		Neurodegeneration	Infections and Inflammatory Diseases
Breast [70,71,79,152,153,157,158,159,165]	Breast [173]	Ataxia telangiectasia [117,221]	CMV [209]
Colon [220]	Lung [72,134,171]	Amyotrophic lateral sclerosis [77,81,196]	Ebola virus [128,129]
Esophageal [163]	Acute promyelocytic leukemia [73]	Parkinson’s disease [133]	Inflammatory diseases [216,218,219,220,221,222,223,224,225]
Hepatic [164]		Traumatic brain injury [81]	Influenza A and B [67,124,125,208]
Nasopharyngeal [168]			Hepatitis C [213,214]
Pancreatic [166,167]			HIV [202,203,204,205]
Prostate [161]			HPV16 pseudovirus [207]
			Listeria monocytogenes [206]
			Mendelian susceptibilities to mycobacterial disease [33,34,78]
			SARS-CoV-2 virus [145,199,200]
			Tuberculosis [217]
			Type I interferonopathies [34,78,82,83,84,215,219]
**The Role of ISG15**			
Antagonizes protein turnover Promotes tumor progression and invasion by disrupting cellular architecture and stabilizing the oncoproteins	Inhibits tumorigenesis by destabilizing growth regulatory proteins Modulates immune system functions	Impairs protein turnover Increases basal autophagy	Inhibits viral budding and release Increases infection-induced autophagy Mediates inflammation-induced vascular damage

## Data Availability

Not applicable.
